# Analysis of Postoperative Quality of Life in Patients With Acute Type A Aortic Dissection After Extensive Arch Repair via Partial Upper Sternotomy

**DOI:** 10.31083/RCM50476

**Published:** 2026-09-11

**Authors:** Linfeng Xie, Xiujuan Wang, Zhaofeng Zhang, Debin Jiang, Xinfan Lin

**Affiliations:** ^1^Department of Cardiovascular Surgery, Fujian Medical University Union Hospital, 350001 Fuzhou, Fujian, China; ^2^Key Laboratory of Cardio-Thoracic Surgery, Fujian Province University, 350001 Fuzhou, Fujian, China; ^3^Fujian Provincial Center for Cardiovascular Medicine, 350001 Fuzhou, Fujian, China

**Keywords:** acute type A aortic dissection, quality of life, extensive total arch repair, partial upper sternotomy, follow-up

## Abstract

**Background:**

This study aimed to compare health-related quality of life (HRQoL) in patients with acute type A aortic dissection (AAAD) who underwent extensive arch repair via partial upper sternotomy (PUS) or full median sternotomy (FS).

**Methods:**

Data were collected from 112 patients with AAAD who were divided into two groups based on the surgical approach. HRQoL was assessed using the MOS 36-item Short-Form Health Survey (SF-36) at discharge (AD), the third postoperative month (POM3), and the twelfth postoperative month (POM12), whereas anxiety symptoms were evaluated using the Spielberger State-Trait Anxiety Inventory state and trait scores (STAI-S and STAI-T) only at POM3.

**Results:**

The PUS group had a numerically lower rate of sternal wound complications than the FS group (1.8% vs. 8.8%; *p* = 0.206), with no significant differences in other in-hospital events. Overall, 112 patients completed the 1-year follow-up. Repeated-measures analyses showed progressive improvement in SF-36 scores from discharge to POM12 in both groups. Compared with the FS group, the PUS group reported better early postoperative HRQoL in selected domains, including role physical, bodily pain, role emotional, and mental health; however, these between-group differences were attenuated at POM12. At POM3, the STAI-S and STAI-T scores were significantly higher in the FS group than in the PUS group.

**Conclusions:**

Partial upper sternotomy for extensive arch repair was feasible and was not associated with an apparent increase in perioperative adverse events. Compared with full sternotomy, PUS was associated with less reported pain, lower psychological burden, and better early postoperative HRQoL in selected patients with AAAD.

## 1. Introduction

Since its first proposal by Svensson in 1997 [1], partial upper sternotomy (PUS) has been used as a minimally invasive access route in selected cardiac and proximal aortic procedures [2]. Nevertheless, because acute type A aortic dissection (AAAD) is a life-threatening emergency with substantial early mortality and long-term morbidity, full median sternotomy (FS) remains the standard approach for extensive aortic repair, as it provides rapid and reliable exposure [3,4]. Previous institutional experience has suggested that extensive repair of AAAD through PUS is technically feasible and may achieve acceptable early clinical outcomes [3]. With continued refinements in minimally invasive cardiac surgery, reduced surgical trauma, improved thoracic stability, less postoperative pain, and better cosmetic results have become increasingly relevant patient-centered outcomes [5].

However, evidence regarding the psychological burden and health-related quality of life (HRQoL) of AAAD patients undergoing extensive arch repair through PUS remains limited. Recent studies and reviews have emphasized that survivors of aortic dissection may experience persistent impairment in physical and mental health, and that HRQoL should be incorporated into postoperative outcome assessment [6,7,8,9,10]. Therefore, we conducted a retrospective cohort study to compare postoperative HRQoL and anxiety symptoms between patients who underwent extensive aortic surgery through either the conventional FS approach or the PUS approach at our institution. In this study, all patients underwent ascending aorta replacement combined with implantation of a modified triple-branched stent graft [11].

## 2. Methods

### 2.1 Study Population

Between June 1, 2020 and June 1, 2021, we conducted a questionnaire survey involving adult patients with AAAD who underwent ascending aorta replacement combined with a modified triple-branched stent graft, performed either via a standard FS approach or a PUS approach. The diagnosis of AAAD was confirmed through computed tomography angiography (CTA) and echocardiography. In this study, all procedures were performed by the same operator and surgical team. The choice of surgical approach was based on the surgeon’s experience. The exclusion criteria were as follows: (1) concomitant mitral or tricuspid valve disease, or coronary artery disease requiring additional surgical intervention; (2) a history of psychiatric disorders; (3) severe comorbidities that could significantly affect quality of life, such as malignant tumors; (4) inability to communicate due to hearing or speech impairment; and (5) refusal to participate in the survey.

The study was approved by the Ethics Committee of Fujian Medical University Union Hospital (Approval No. 2022KY031) and adhered to the Helsinki Declaration. Due to its retrospective nature, approval was granted by the Ethics Committee of Fujian Medical University Union Hospital to waive the requirement for written informed consent. The data were analyzed in an anonymous manner.

### 2.2 Triple-Branched Stent Graft

The triple-branched stent graft was originally developed for extensive primary repair of the thoracic aorta in AAAD [11]. It is a branch-integrated graft composed of a self-expanding nitinol stent framework and polyester vascular graft fabric, including one main stent graft and three side-branch stent grafts.

### 2.3 Operative Procedure

In this study, we used a modified triple-branched stent graft (20173034262, Yuhengjia Technology Company, Beijing, China) that was commercially manufactured in China. The device consists of a main stent graft and three side-branch stent grafts designed for the aortic arch vessels. The proximal portion and the aortic arch segment immediately distal to the three branches are covered only by polyester graft fabric, without a supporting nitinol stent framework in this region.

Patients were placed in the supine position under general anesthesia. A PUS (“L”-shaped) incision was made between the sternal notch and the level of the fourth intercostal space, then extended to the left fourth intercostal space (Fig. 1). The initial skin incision measured approximately 10–15 cm but was extended if surgical exposure proved inadequate. After opening the pericardium, it was fixed to the drapes with several stay sutures, thereby elevating the heart and aortic arch anteriorly, which provided excellent exposure. The establishment of cardiopulmonary bypass typically begins with cannulation of the right axillary and femoral arteries, followed by direct cannulation of the right atrium. Once the nasopharyngeal temperature reached 32 °C, an aortic cross-clamp was applied. Aortic root surgery was performed based on the condition of the aortic root, valve, and coronary ostia. An oblique incision was made near the small bend of the aortic arch. The main segment of the triple-branched stent graft was inserted into the true lumen of the aortic arch and proximal descending aorta. Subsequently, three lateral wall grafts were implanted into the corresponding arcuate vessels in sequence, followed by the release of the stent graft. Finally, the ends of the triple-branch stent were continuously anastomosed to the artificial polyester vessel, thereby completing the operation [3,11].

**Fig. 1.**
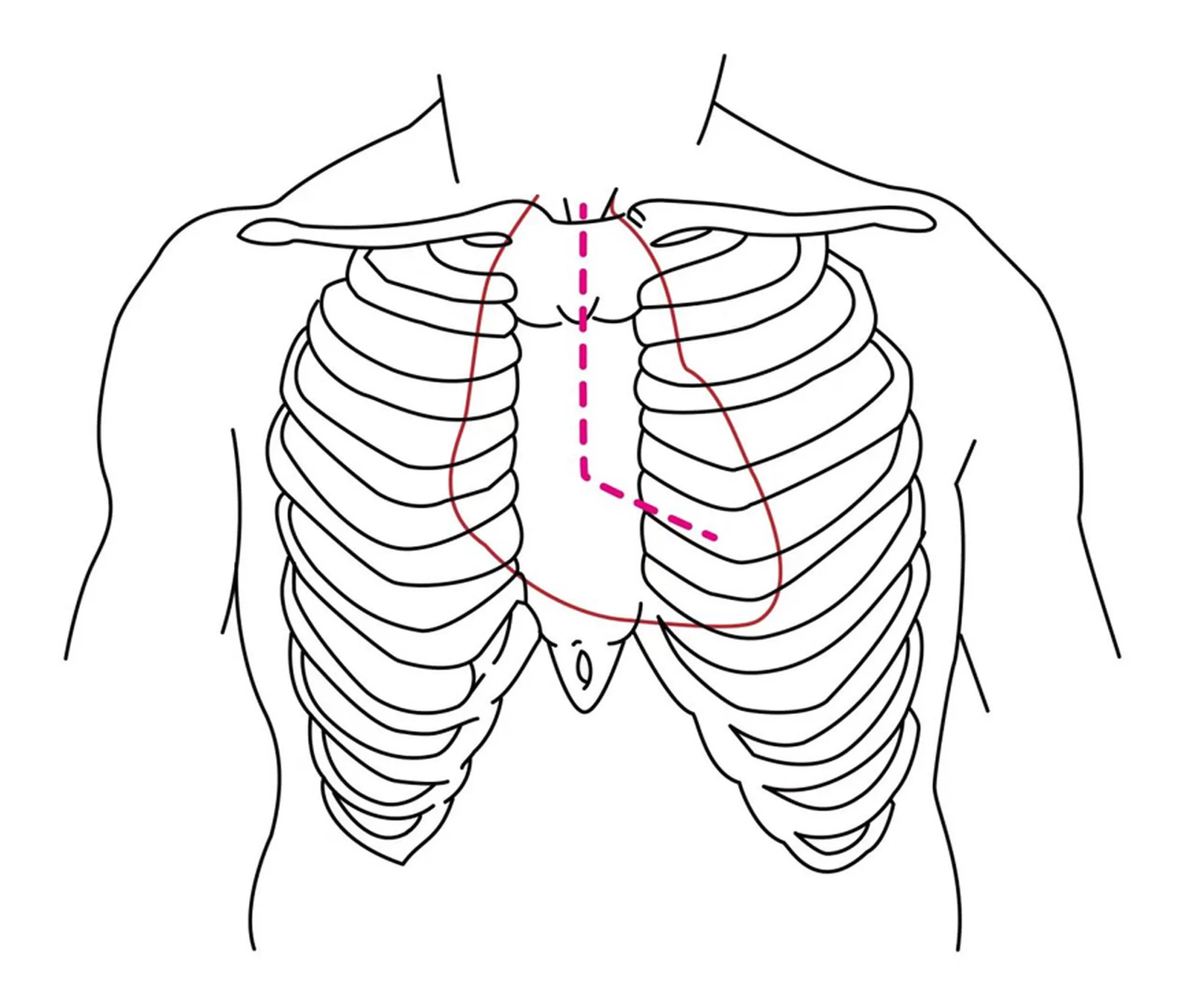
**Partial upper sternotomy incision for extensive arch repair of acute type A aortic dissection**. The magenta dashed line indicates the actual skin and sternal incision, consisting of a partial upper median sternotomy extended into the left fourth intercostal space. The red solid line indicates the surgical exposure area after opening the chest.

### 2.4 Data Collection

Clinical data were extracted from the hospital electronic medical record system and cross-checked by two investigators. The collected variables included preoperative baseline characteristics, operative details, and postoperative outcomes. Postoperative HRQoL was assessed using standardized questionnaires on the morning of discharge, at the third postoperative month, and at the twelfth postoperative month during outpatient follow-up or structured follow-up assessment.

### 2.5 Questionnaire Survey

The assessment tools comprised the Chinese versions of the Medical Outcomes Study (MOS) 36-Item Short-Form Health Survey (SF-36) and the Spielberger State-Trait Anxiety Inventory (STAI). HRQoL was evaluated using the SF-36, a generic and widely used instrument for assessing health status across different clinical populations [12,13]. The SF-36 contains 36 items grouped into eight domains: physical functioning, role physical, bodily pain, general health, vitality, social functioning, role emotional, and mental health. A higher domain score indicates better perceived health status.

The Spielberger STAI, including both state anxiety (STAI-S) and trait anxiety (STAI-T), was used to characterize anxiety symptoms and was administered only at POM3. The STAI-S reflects transient anxiety related to a stressful situation, whereas the STAI-T reflects a more stable individual tendency toward anxiety [14,15,16]. The total score ranges from 20 to 80, with higher scores indicating more severe anxiety symptoms. In previous cardiovascular and clinical populations, a score of 40 or higher has commonly been used as a threshold indicating clinically relevant anxiety symptoms [17].

### 2.6 Statistical Analysis

Statistical analysis was performed using SPSS software version 22.0 (SPSS Inc., Chicago, IL, USA). The distribution of continuous variables was assessed before between-group comparisons. Normally distributed variables are presented as mean ± standard deviation and were compared using Student's *t*-test, whereas non-normally distributed variables are presented as median [interquartile range] and were compared using the Mann-Whitney *U* test. Categorical variables are expressed as numbers and percentages and were compared using the chi-squared test or Fisher's exact test, as appropriate according to expected cell counts. For SF-36 outcomes assessed repeatedly at discharge, POM3, and POM12, longitudinal analyses were performed using linear mixed-effects models. Because these HRQoL analyses involved multiple domains and were exploratory in nature, the findings were interpreted with caution rather than as definitive confirmatory evidence. Spearman's rank correlation coefficient was used to assess associations between STAI-S scores and SF-36 domain scores at POM3. All tests were two-sided, and *p* < 0.05 was considered statistically significant.

## 3. Results

### 3.1 Baseline Characteristics

A total of 112 patients met the inclusion criteria and were initially enrolled. During the index hospitalization, 4 patients in the PUS group and 5 patients in the FS group died. Four additional patients did not complete follow-up because of severe neurological sequelae, postoperative low cardiac output syndrome-related death after discharge, or loss to follow-up following accidental death. Finally, 112 patients completed the 1-year follow-up and were included in the HRQoL analysis (55 patients in the PUS group and 57 patients in the FS group, with no conversions). The baseline characteristics are presented in Table 1. No statistically significant differences were observed in measured preoperative characteristics between the two groups.

**Table 1.** **Preoperative characteristics of patients**.

Items	Total (n = 112)	PUS Group (n = 55)	FS Group (n = 57)	*p* value
Age (years)	51.0 ± 11.6	50.4 ± 11.4	51.6 ± 11.8	0.592
Male (n, %)	58 (51.8)	29 (52.7)	29 (50.9)	0.845
BMI (kg/m^2^)	25.8 ± 1.6	25.8 ± 1.6	25.9 ± 1.7	0.689
Hypertension (n, %)	87 (77.7)	40 (72.7)	47 (82.5)	0.218
Diabetes mellitus (n, %)	5 (4.5)	2 (3.6)	3 (5.3)	0.678
Renal dysfunction^ a ^(n, %)	18 (16.1)	8 (14.5)	10 (17.5)	0.667
COPD (n, %)	2 (1.8)	1 (1.8)	1 (1.8)	0.980
Marfan syndrome (n, %)	6 (5.4)	5 (9.1)	1 (1.8)	0.086
Pericardial effusion ^b^ (n, %)	4 (3.6)	2 (3.6)	2 (3.5)	0.971
Acute aortic regurgitation (n, %)	25 (22.3)	13 (23.6)	12 (21.1)	0.744
Malperfusion syndromes ^c^ (n, %)	2 (1.8)	1 (1.8)	1 (1.8)	0.980
Preoperative LVEF (%)	63.0 ± 7.1	64.4 ± 5.8	61.7 ± 7.9	0.207

Data are presented as mean ± standard deviation, median [interquartile range] or number (%). Chi-square test for categorical variables and *t*-test or Wilcoxon rank-sum test for continuous variables. Preoperative LVEF was not normally distributed and was therefore compared using the Mann–Whitney *U* test rather than *t*-test. BMI, body mass index; COPD, chronic obstructive pulmonary disease**; **LVEF, left ventricular ejection fraction; PUS, partial upper sternotomy; FS, full median sternotomy. a: Renal dysfunction, preoperative serum creatinine >130 μmol/L. b: Pericardial effusion, intraoperatively confirmed effusion volume exceeding 300 mL and based on cardiac color ultrasonography, an anechoic area in the left ventricular posterior wall was increased (>10 mm). c: Malperfusion syndromes, clinical symptoms or evidence of ischaemia involving three or more organs of the heart, brain, spinal, liver, spinal cord, or gastrointestinal tract and extremities.

### 3.2 Intraoperative and Postoperative Results

The detailed intraoperative and postoperative results are presented in Table 2. The rate of sternal wound complications was numerically lower in the PUS group than in the FS group (1.8% vs. 8.8%, *p* = 0.206 by Fisher’s exact test), but this difference did not reach statistical significance. No statistically significant differences were observed between the two groups in terms of bleeding events, acute kidney injury, reintubation, hepatic insufficiency, nervous system complications, or low cardiac output syndrome.

**Table 2.** **Intraoperative and postoperative data of patients**.

Items	Total (n = 112)	PUS Group (n = 55)	FS Group (n = 57)	*p* value
Intraoperative				
Total operation time (min)	303.1 ± 79.6	306.1 ± 88.7	300.2 ± 70.5	0.406
CPB time (min)	146.6 ± 56.0	151.1 ± 62.3	142.3 ± 49.2	0.701
ACC time (min)	51.5 ± 28.2	51.2 ± 24.5	51.8 ± 31.4	0.518
SCP time (min)	14.6 ± 3.5	14.5 ± 3.9	14.8 ± 3.0	0.458
DHCA time (min)	2.9 ± 2.2	3.0 ± 2.7	2.8 ± 1.6	0.223
Bentall procedure	80	38	42	0.591
Valve-sparing root replacement	23	13	10	0.425
Ascending aorta replacement	9	4	5	0.770
Postoperative				
Mechanical ventilation time (h)	66.5 ± 69.1	62.7 ± 70.7	70.1 ± 68.0	0.573
ICU stay time (day)	5.6 ± 8.4	5.4 ± 8.3	5.8 ± 8.6	0.782
Postoperative hospital stays (d)	16.4 ± 9.6	15.5 ± 9.2	17.3 ± 9.9	0.305
Bleeding event ^d^	1 (0.9)	1 (1.8)	0	0.491
Reintubation (n, %)	2 (1.8)	0	2 (3.5)	0.496
Acute kidney injury ^e ^(n, %)	21 (18.8)	9 (16.4)	12 (21.1)	0.630
Hepatic insufficiency ^f ^(n, %)	27 (24.1)	13 (23.6)	14 (24.6)	1.000
Nervous system complications ^g^ (n, %)	7 (6.3)	3 (5.5)	4 (7.0)	1.000
Low cardiac output syndrome ^h ^(n, %)	1 (0.9)	1 (1.8)	0	0.491
Sternal wound complications ^i ^(n, %)	6 (5.4)	1 (1.8)	5 (8.8)	0.206

Data are presented as mean ± standard deviation, median [interquartile range] or number (%). Continuous variables were compared using the *t* test or Wilcoxon rank sum test, as appropriate. Categorical variables with small expected cell counts were compared using Fisher’s exact test. CPB, cardiopulmonary bypass; ACC, aortic cross-clamp; SCP, selective cerebral perfusion; DHCA, deep hypothermic circulatory arrest; ICU, intensive care unit; CRRT, continuous renal replacement therapy. d: Bleeding event, re-exploration for postoperative bleeding. e: Acute kidney injury, defined as 50% rise in baseline creatinine or new need for CRRT. f: Hepatic insufficiency, defined as bilirubin greater than 5 mg/dL persisting for more than 5 days postoperatively. g: Nervous system complications, unconsciousness, delirium, coma and other temporary neurological dysfunctions; cerebral infarction; paraplegia or paraparesis. h: Low Cardiac Output Syndrome, the need for two or more inotropic medications to maintain systolic blood pressure greater than 90 mmHg or cardiac output >2.2 L/min/m^2^ after adjusting preload and correcting all electrolyte or blood gas abnormalities. i: Sternal wound complications, sternal instability after a sternotomy can be responsible for delayed wound healing or the development of sternal infections.

### 3.3 Questionnaire

Fig. 2 presents the longitudinal changes in SF-36 scores from discharge to postoperative month 12 (POM12) in the PUS and FS groups. In the repeated-measures analysis using linear mixed-effects models, a significant time effect was observed across all eight SF-36 domains, indicating gradual improvement in HRQoL after discharge in both groups. No significant group-by-time interaction was observed for physical functioning, general health, vitality, or social functioning, suggesting broadly comparable recovery trajectories between the two groups in these domains. In contrast, the PUS group showed better early postoperative scores in selected domains, particularly role physical, bodily pain, role emotional, and mental health. Model-based comparisons showed that the PUS group had higher role physical scores at discharge, higher bodily pain and role emotional scores at discharge and POM3, and higher mental health scores at discharge and POM3. These between-group differences became attenuated by POM12, suggesting that the HRQoL advantage associated with PUS was most evident during the early postoperative recovery period.

**Fig. 2.**
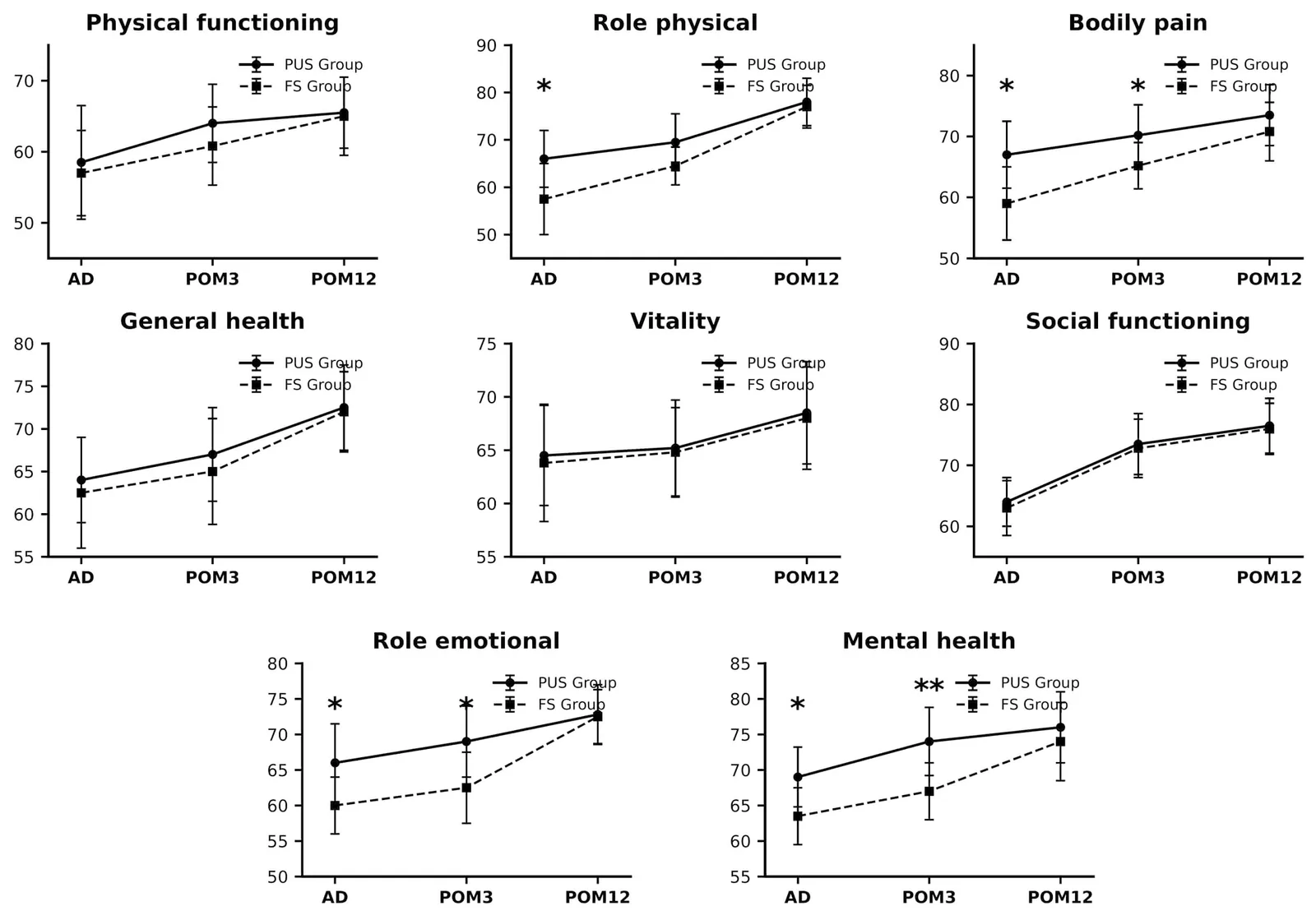
**The comparison of the SF-36 scores between groups at different time points**. AD, At discharge; POM3, The third postoperative month; POM12, The twelfth postoperative month. *: PUS Group vs FS Group (*p* < 0.05), **: PUS Group vs FS Group (*p* < 0.01).

The STAI scores were assessed in both groups at the third postoperative month (Table 3). Mean STAI-S and STAI-T scores exceeded the commonly used threshold of 40 in both groups, suggesting a considerable anxiety burden after AAAD surgery. The FS group demonstrated significantly greater anxiety levels than the PUS group, as evidenced by higher STAI-S scores (49.1 ± 12.3 vs. 42.5 ± 10.1, *p* < 0.01) and STAI-T scores (51.4 ± 9.8 vs. 44.2 ± 8.3, *p* < 0.01).

**Table 3.** **Assessment of different level of anxiety at POM3**.

Items	PUS Group (n = 55)	FS Group (n = 57)	*p* value
STAI-S	42.5 ± 10.1	49.1 ± 12.3	<0.01
STAI-T	44.2 ± 8.3	51.4 ± 9.8	<0.01

Data are presented as mean ± standard deviation. POM3, the third postoperative month; STAI-S, state anxiety subscale in the Spielberger State-Trait Anxiety Inventory; STAI-T, trait anxiety subscale in the Spielberger State-Trait Anxiety Inventory.

Spearman’s rank correlation analysis was conducted between the SF-36 domain scores and STAI-S scoresat POM3 (Fig. 3). STAI-S scores showed strong negative correlations with mental health and role emotional scores (r = –0.827 and r = –0.692, respectively; both *p* < 0.001). Moderate negative correlations were also observed for bodily pain and vitality, while weaker but statistically significant negative correlations were observed for physical functioning and general health (with all *p* < 0.05). These findings suggest that anxiety symptoms were closely associated with poorer mental health, emotional role functioning, pain-related quality of life, and selected physical health domains.

**Fig. 3.**
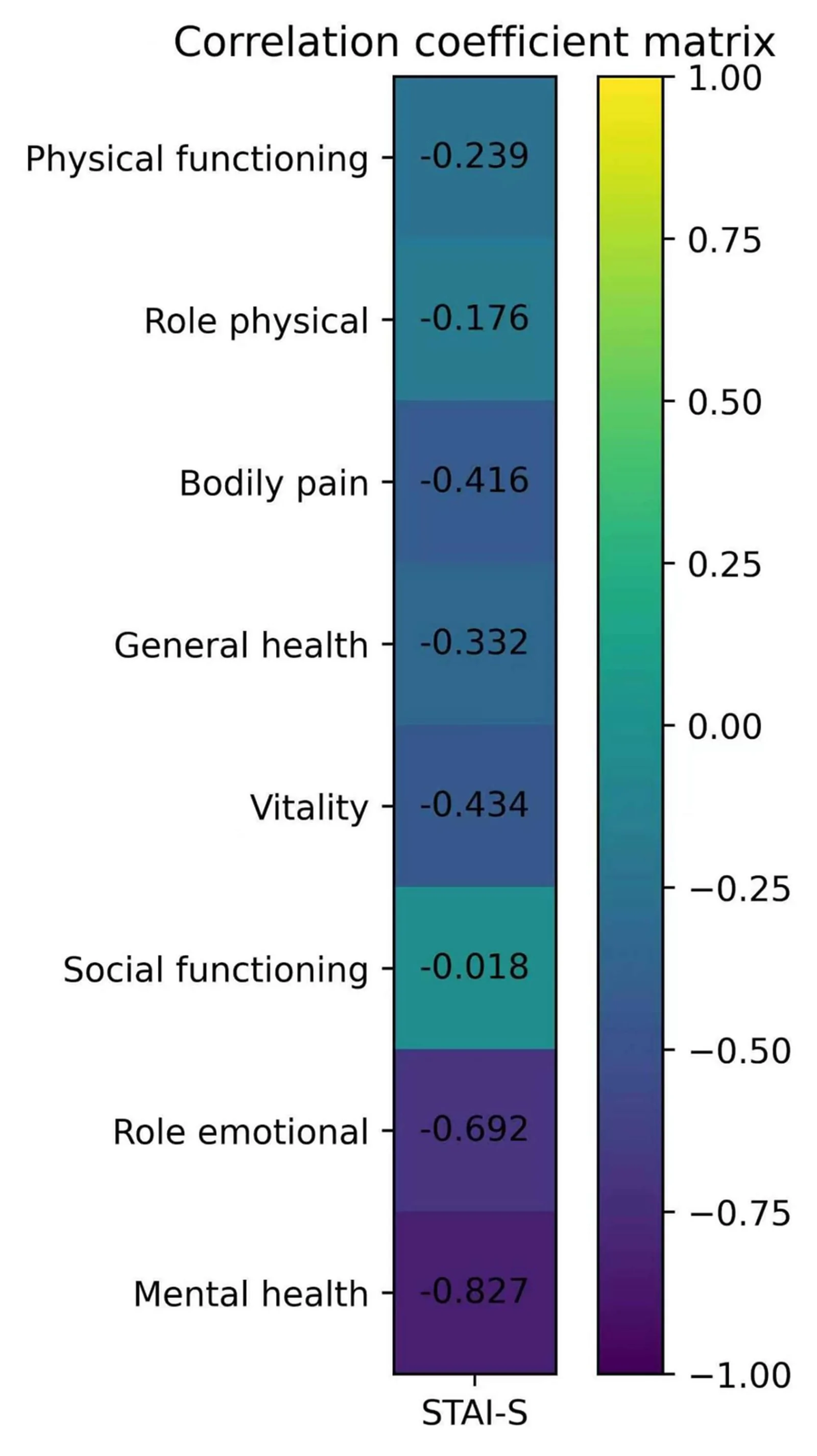
**Correlation coefficient matrix between STAI-S and SF-36 subdomains**.

## 4. Discussion

Surgery for AAAD remains one of the most complex and challenging scenarios in adult cardiac surgery. Although advances in diagnosis, operative strategy, and perioperative management have improved survival, AAAD survivors may still experience substantial morbidity and impaired long-term physical and mental well-being [4,6,7]. Recent HRQoL studies in aortic dissection have shown that postoperative recovery cannot be fully captured by mortality or major complications alone, as persistent pain, functional limitation, anxiety, depression, and post-traumatic stress symptoms may continue to affect patients after discharge [6,7,8,9,10,18,19]. In this context, patient-centered outcomes are increasingly important when evaluating surgical approaches for AAAD.

This study aimed to characterize the HRQoL of AAAD patients after extensive aortic surgery and to explore whether the surgical access route was associated with early postoperative patient-reported outcomes. Using the SF-36, we compared HRQoL outcomes between patients treated through FS and those treated through PUS at discharge, POM3, and POM12; anxiety symptoms were assessed using the STAI only at POM3. This design allowed us to examine not only conventional perioperative safety but also recovery dimensions that are highly relevant to patients.

In our study, SF-36 scores in both groups were relatively low at discharge and improved gradually during follow-up, consistent with the major physiologic and psychological burden imposed by AAAD and emergency surgery. Compared with the FS group, the PUS group had higher scores in role physical at discharge, bodily pain at discharge and POM3, role emotional at discharge and POM3, and mental health at discharge and POM3. These findings are consistent with broader evidence from minimally invasive cardiac surgery suggesting that less invasive access may accelerate early recovery and improve short-term patient-reported outcomes [5,20,21]. The PUS approach, with a shorter incision and reduced sternal disruption, may reduce pain and improve patients’ confidence during coughing, deep breathing, ambulation, and early rehabilitation. By POM12, between-group differences became less apparent, suggesting that the advantage of PUS may be most prominent during early recovery.

The World Health Organization (WHO) has long defined health as a state of physical, mental, and social well-being rather than merely the absence of disease [22], and the World Health Organization Quality of Life (WHOQOL) framework further emphasizes the subjective and multidimensional nature of quality of life [23]. Medical trauma and life-threatening cardiovascular events can lead to anxiety, depression, and post-traumatic stress symptoms [10,18]. In the present cohort, at POM3, both groups had mean STAI scores above the commonly used threshold for clinically relevant anxiety, indicating that psychological distress is common after AAAD surgery and should not be overlooked in postoperative care.

Several factors may explain the lower STAI scores observed in the PUS group. First, the FS approach involves a longer midline incision and more extensive sternal trauma, which may increase pain, restrict activity, and make patients more aware of the severity of the operation. Second, persistent postoperative pain can interfere with sleep, rehabilitation, and daily function, thereby amplifying anxiety symptoms. Third, improved body image and earlier functional recovery after a smaller incision may reduce perceived illness burden. Although these explanations remain hypothesis-generating, they support the need to incorporate psychological screening and patient-reported outcomes into routine follow-up after AAAD repair.

The influence of pain intensity and incision-related concerns on HRQoL is often underestimated in cardiac surgery. Previous studies have reported persistent pain after sternotomy and have shown that pain can affect mood, daily activity, and postoperative functioning [20,24,25]. Our study found higher bodily pain scores in the PUS group at discharge and POM3, suggesting less pain-related impairment during early recovery. This may be attributable to less tissue disruption, a smaller scar, fewer sternal wires, and reduced risk of sternal instability. Improved pain control may also facilitate participation in breathing exercises and cardiac rehabilitation, although this hypothesis requires confirmation in prospective studies.

## 5. Limitations

First, it was a retrospective single-center study with a modest sample size. Because the choice of surgical approach was based on the surgeon’s experience rather than random allocation, selection bias cannot be excluded. Although measured baseline variables were broadly comparable between groups, unmeasured differences in anatomic complexity, frailty, socioeconomic factors, education level, self-care ability, rehabilitation adherence, and baseline psychological status may have influenced postoperative HRQoL [24]. Second, because AAAD is an acute life-threatening emergency, preoperative baseline HRQoL and anxiety data were unavailable. Therefore, this study evaluated absolute postoperative scores rather than within-patient changes from baseline. Third, the analyses of multiple SF-36 domains at several time points were exploratory, and the study was not powered for all individual HRQoL domains. Fourth, because the STAI was assessed only at POM3, longitudinal changes in anxiety could not be evaluated. Fifth, the follow-up period was limited to 1 year; longer follow-up is needed to determine whether the early HRQoL advantage associated with PUS persists over time. Finally, the findings should be validated in multicenter prospective cohorts or randomized studies with prespecified statistical adjustment, such as multivariable modeling or propensity score methods.

## 6. Conclusions

In selected AAAD patients undergoing extensive arch repair, the PUS approach was feasible and showed no apparent increase in perioperative adverse events compared with FS. Patients treated with PUS reported better early postoperative HRQoL, particularly in pain-related, emotional, and mental health domains, and lower anxiety scores at POM3. These potential advantages may be related to reduced surgical trauma and improved subjective recovery. Given the retrospective and non-randomized design, these findings should be interpreted as hypothesis-generating and require confirmation in larger prospective studies.

## Data Availability

The data from this study will not be shared publicly. All data included in this study are available upon request by contact with the corresponding author.

## Abbreviations

PUS, partial upper sternotomy; FS, full median sternotomy; HRQoL, health-related quality of life; AAAD, acute type A aortic dissection; SF-36, the MOS 36-item Short-Form Health Survey; STAI-S and STAI-T, the Spielberger State-Trait Anxiety Inventory state and trait scores; AD, the time point at discharge; POM3, the third postoperative month; POM12, the twelfth postoperative month.
